# Comparative genomics reveals the emergence of copper resistance in a non‐pigmented *Xanthomonas* pathogen of grapevine

**DOI:** 10.1111/1758-2229.13164

**Published:** 2023-05-30

**Authors:** Rekha Rana, Gagandeep Jaiswal, Kanika Bansal, Prabhu B. Patil

**Affiliations:** ^1^ Bacterial Genomics and Evolution Laboratory CSIR‐Institute of Microbial Technology Chandigarh India; ^2^ The Academy of Scientific and Innovative Research Ghaziabad India

## Abstract

*Xanthomonas citri* pv. *viticola* (*Xcv*) is the causal agent of bacterial canker in grapevine. The pathogen is restricted to India, where it was first reported in the 1970s, and Brazil. In the present study, we report the first complete genome sequence of *Xcv* LMG965, which is a reference pathotype strain. We also report genome sequences of additional isolates from India and comparative genome‐based studies of isolates from Brazil. Apart from revealing the monophyletic origin of the pathovar, we could also confirm a common frameshift mutation in a gene that is part of the Xanthomonadin pigment biosynthetic gene cluster in all the isolates. The comparative study also revealed multiple intrinsic copper resistance‐related genes in Brazilian isolates, suggesting intense selection, possibly because of heavy and indiscriminate usage of copper as an antimicrobial agent in the orchards. There is also the association of a Tn3‐like transposase in the vicinity of the copper resistance genes, indicating a potential for rapid diversification through horizontal gene transfer events. The findings, along with genomic resources, will allow for systematic genetic and functional studies of *Xcv*.

## INTRODUCTION

Grapevine bacterial canker of grapes (*Vitis vinifera* L.) is caused by *Xanthomonas citri* pv. *viticola* (*Xcv*) (da Gama et al., 2018). The disease leads to the development of cankers on almost all plant parts, including leaves, berries, petioles and twigs. Apart from *Vitis vinifera*, the pathogen is also reported to infect *Azadirachta indica* (neem), *Phyllanthus maderaspatensis*, cashew plants, *Mangifera indica* (mango) and plants of *Amaranthus* and *Glycine* genus (Chand & Kishun, 1990; da Gama et al., 2018; Nayudu, 1972; Peixoto et al., 2007). The disease was first reported in India in 1972 with disease outbreaks in the 1980s (Chand & Kishun, 1990). The disease was endemic to India until 1998, when *Vitis vinifera* plants in Brazil were reported to be developing a similar disease, and the concerning bacterial strains were found to be comparable to the Indian pathotype strain, NCPPB 2475 (Trindade et al., 2007). There is also a report of its presence from Thailand in the Asian region, and the disease is not yet reported from the European Union and other grape‐growing areas (Health et al., 2021; Midha & Patil, 2014). Recently, the pathogen was assessed by the European Food Safety Authority as a potential quarantine threat (Health et al., 2021).

The formation of yellow‐coloured colonies due to the presence of Xanthomonadin pigment is a characteristic feature of the *Xanthomonas* group of phytopathogens (He et al., 2020). However, few pathogens have acquired mutations in the Xanthomonadin biosynthesis gene cluster, like frameshift mutation, deletion and insertion, to lose their pigmentation (Midha & Patil, 2014). *X. citri* pv. *viticola* (*Xcv*) is one such pathovar that forms white colonies. Previously (Midha & Patil, 2014), a frameshift mutation in a gene encoding phosphotransferase/dehydratase, resulting in a truncated protein was reported to be the reason behind this phenotype. Interestingly, in another pathovar, *X. citri* pv. *mangiferaeindicae* (*Xcm*) of this species, that infects mango, the pigment cluster is deleted (Midha & Patil, 2014). Xanthomonadin pigment is reported to be important for the protection of the bacterium from photodamage but not required for virulence (He et al., 2020). It is possible that after acquiring a frameshift mutation in one of the genes, over time, the pathovar loses the pigment cluster. Hence, there is a need to confirm the mutation status over decades in multiple strains of diverse geographic locations. However, such studies are lacking in *Xcv*, *Xcm*, and in other *Xanthomonas* pathogens that produce white‐coloured colonies. At the same time, it is also important to understand why few of the *Xanthomonas* pathogens are acquiring mutations in the Xanthomonadin gene cluster or pigment genes.

Copper‐based bactericides have been frequently used for plant disease management for years. Extensive use of these bactericides has resulted in the resistance to copper compounds in phytopathogens (Behlau et al., 2011; Cooksey, 1990; Xiaojing et al., 2022). Copper sprays are also used for the control and prevention of grapevine bacterial canker (Malavolta Jr et al., 1999; Marques et al., 2009). There has been a constant increase in copper tolerance over the years 1998–2006 in *X. citri* pv. *viticola* strains (Marques et al., 2009). In earlier studies, multiple genetic mechanisms of many copper‐resistant strains from different *Xanthomonas spp*. have been reported (Basim et al., 2005; Behlau et al., 2011; Giovanardi et al., 2016; Lee et al., 1994). Three of them are plasmid‐borne, while one is chromosomally encoded (Basim et al., 2005; Huang et al., 2021). Huang et al. characterised *Xanthomonas*‐associated copper resistance gene clusters into three groups, however, the genomic determinants of copper resistance/homeostasis in *X. citri* pv. *viticola* are not yet reported. Hence, it will be interesting and important to investigate the origin and evolution of copper resistance loci over time in different geographic regions. The complete genome resource, along with genome sequences of multiple strains from diverse regions, will help in the systematic study of pathogenicity and management.

## EXPERIMENTAL PROCEDURES

### Genome sequencing, assembly and annotation

*X. citri* pv. *viticola* strains LMG965, LMG966 and LMG967 were acquired from the Belgium Co‐ordinated Collection of Microorganisms (BCCM). Cultures were grown overnight in a Nutrient Broth medium, and their genomic DNA was isolated using the Quick‐DNA™ Fungal/Bacterial Miniprep Kit (Zymo Research, Irvine, CA). Genomic DNA quality and quantity were assessed using the Nanodrop 1000 (Thermo Fisher Scientific) and Qubit 2 fluorometer (Invitrogen; Life Technologies), respectively. Raw reads for the draft genome of LMG965 were obtained from the Illumina Nova‐Seq platform facility at MedGenome (Hyderabad, India). LMG966 and LMG967 were sequenced using an in‐house Illumina MiSeq platform using the Nextera XT sample preparation kit with Illumina paired‐end sequencing libraries (250*2, read length) (Illumina, Inc., San Diego, CA, USA). The raw reads were quality assessed using FastQC version 0.11.9 (https://qubeshub.org/resources/fastqc). Raw reads were filtered using Trim‐galore v0.6.7 (Babraham Bioinformatics), and de novo assembled using SPAdes v3.13.0 (Prjibelski et al., 2020). For long‐read sequencing of LMG965, a ligation sequencing kit (SQK‐LSK109) was used for library preparation. All the bead‐washing steps were performed using AMPure beads (Beckman Coulter). Native barcoding and adaptor ligation steps were performed as per the protocols given by Oxford Nanopore Technologies using Native barcoding kit (EXP‐NBD104). Finally, 13 μL of the prepared DNA library was sequenced using the MinION R9 flow cell (FLO‐MIN106) with MinKNOW software v3.6.5 for 72 h (http://community.nanoporetech.com Oxford Nanopore Technologies). FAST5 reads were base called using the guppy v4.2.2+effbof8 software (http://community.nanoporetech.com) and filtered using Filtlong v0.20 (https://github.com/rrwick/Filtlong). Hybrid assembly of filtered long‐reads of LMG965 with Illumina short reads was carried out using Unicycler v0.4.8 (Wick et al., 2017), followed by multiple rounds of polishing using Pilon v1.22 (Walker et al., 2014). Completeness/contamination was estimated using CheckM v1.0.13 (Parks et al., 2015). Quast v5.0.2 was used to assess the genome assembly quality and calculate genome coverage (Gurevich et al., 2013). The genomes were annotated using the Prokaryotic Genome Annotation Pipeline of the National Center for Biotechnology Information (NCBI) (https://www.ncbi.nlm.nih.gov/genome/annotation_prok/).

### *Complete genome features of* Xcv *LMG965*

The circular representation of the chromosome and plasmid of *Xcv* LMG965 was created using the CGview comparison tool (CCT) (Grant et al., 2012). The coding sequences (CDSs) were categorised into clusters of orthologous groups (COGs) using CCT (Grant et al., 2012). Insertion sequence (IS) elements were predicted using ISsaga 2.0 (Varani et al., 2011). Transcription activator‐like effectors (TALEs) were predicted and assigned to TALE classes using the AnnoTALE version 1.5 (Grau et al., 2016).

### Phylogenetic and taxonomic analysis

Genomes of *X. citri* pathovars were retrieved from the NCBI GenBank database for phylogenetic analysis. *X. perforans* DSM18975^T^ was taken as an outgroup. Only genomes with completeness above 95% were taken into consideration and screened using CheckM (Parks et al., 2015). Prokka v1.46 was used to generate GFF files, which were further used as input for Roary v3.13.0 to generate core gene alignment files (Page et al., 2015; Seemann, 2014). A maximum likelihood phylogenetic tree was constructed using PhyML v3.3 with a GTR nucleotide model of substitution with 1000 bootstraps (Guindon et al., 2010). The phylogeny was visualised using iTOL v6 (Letunic & Bork, 2021). The Ortho Average Nucleotide values (orthoANI) were calculated using oANI using USEARCH v11.0.667 (Lee et al., 2016).

### Xanthomonadin pigment cluster analysis

XAC_RS20620 (gene encoding phosphotransferase) from *X. citri* pv. *citri* 306 (NC_003919) was used to retrieve homologous sequences from the *Xcv* strains using standalone tBLASTn. The nucleotide sequences were annotated with the help of ORF Finder to obtain amino acid sequences. Both the amino acid and nucleotide sequences were aligned using MUSCLE inbuilt in MEGA X v10.2.6 (Kumar et al., 2018).

### Pangenome and copper resistome analysis

The pangenome was carried out using Roary v3.13.0 (Page et al., 2015), taking Prokka v1.46 (Seemann, 2014) generated GFF files as input. The homologues of genes involved in copper resistance and tolerance mechanisms were predicted using standalone tBLASTn v2.9.0+. The nucleotide identity between copper resistance/homeostasis‐associated genes from various strains with the *Xcv* strains was calculated using standalone BLASTn v2.9.0+. Copper operon clusters were generated using clinker v0.0.23 (Gilchrist & Chooi, 2021).

## RESULTS

### *Complete genome sequence of the pathovar reference strain of* X. citri *pv.* viticola

**FIGURE 1:**
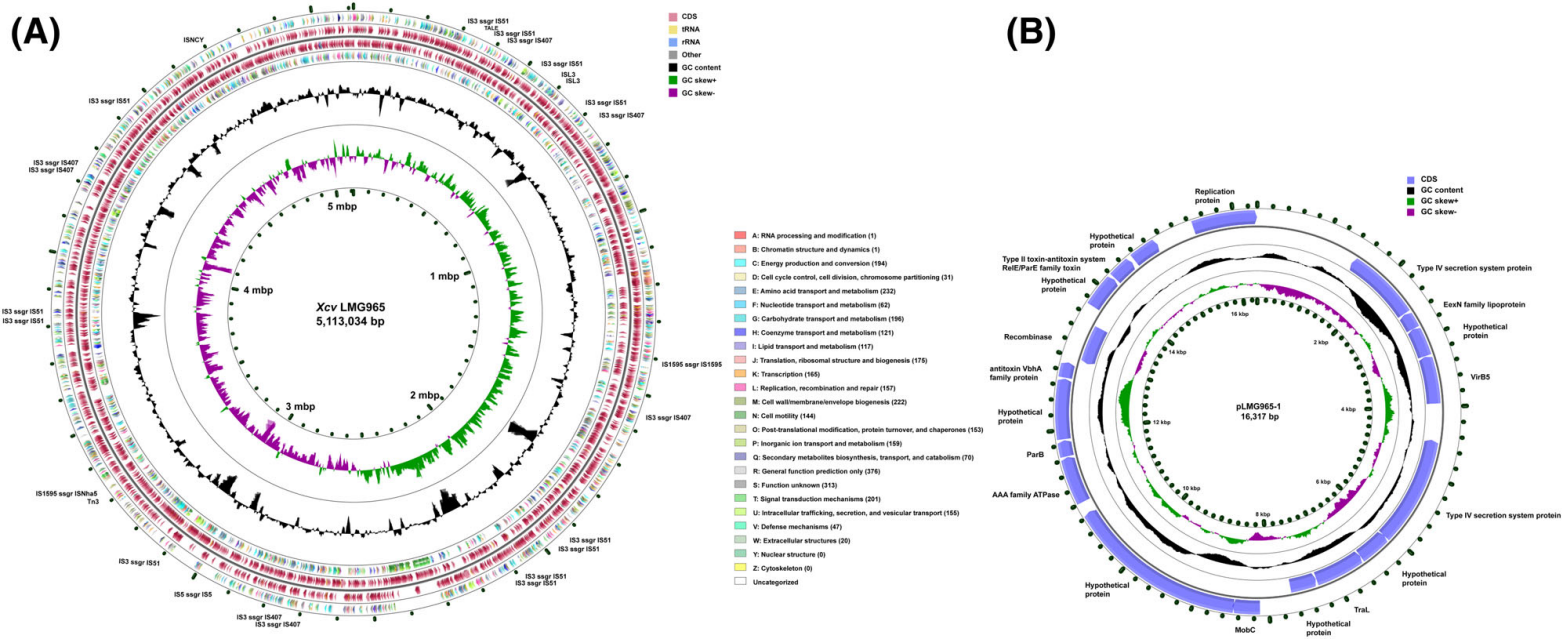
(A) Circular representation of the chromosome of *Xanthomonas citri* pv. *viticola* (*Xcv*) LMG965. The outermost ring depicts coding sequences on the forward strand assigned to clusters of orthologous group (COG) categories. The following two rings show coding sequences and RNAs on forward and reverse strands, respectively. The next ring shows coding sequences on the reverse strand assigned to COG categories. Furthermore, the two innermost rings depict GC content and GC skew (+ and −). The colour coding for all the features is given in the figure. IS elements and transcription activator‐like effector (TALE) coding gene locations are labelled on the outermost ring. (B) Circular representation pLMG965‐1 plasmid of *Xcv* LMG965. The two outermost rings depict CDSs on the forward and reverse strands, followed by two innermost rings depicting GC content and GC skew (+ and −). The colour coding for all the features is given in the figure. CDSs are annotated on the outermost ring.

The complete genome sequence of the pathovar reference strain first isolated from India is depicted in Figure 1. Hybrid assembly with Illumina short reads and Oxford nanopore reads of LMG965 resulted in a circular chromosome of 5.11 Mb and a plasmid of 16.3 kb (Figure 1). The complete genome of *Xcv* LMG965 encodes 4270 coding sequences (CDSs) and 53 tRNAs with two rRNA operons. These coding sequences were further classified into COG categories (Figure 1A). The COG categories with maximum representation were amino acid transport and metabolism (E), cell wall/membrane/envelope biogenesis (M), signal transduction mechanisms (T), carbohydrate transport and metabolism (G), Translation, ribosomal structure and biogenesis (J) and replication, recombination and repair (L). A large number of CDSs were assigned to general function prediction only (R) and function unknown (S) categories (Figure 1A). The genome carries 26 IS elements, further classified into IS element families. The maximum number of IS elements were assigned to IS3 ssgr IS51 and IS3 ssgr IS407 IS element families. IS elements were also assigned to ISL3, IS1595 ssgr IS1595, ISNCY, IS1595 ssgr ISNha5, Tn3 and IS5 ssgr IS5 families (Table S1, Figure 1A).

The complete genome also carries one transcriptional activator‐like effector (TALE) that was assigned to TalJE class by AnnoTALE version 1.5 (Figure 1A, Table S1 **)** (Grau et al., 2016). TALEs are 33–35 amino acid‐long repeats directed to bind host DNA in a sequence‐specific manner. They play an imperative role in virulence by manipulating host gene expression. Usually, in the case of *X. citri*, TALEs are also found to be associated with the plasmids, however, for *Xcv* LMG965, no TALEs were encoded on the plasmid (da Silva et al., 2002; Rana et al., 2022). The plasmid pLMG965‐1 encodes various type IV secretion system‐related proteins, partition, replication and mobilisation proteins, toxin/antitoxin system and hypothetical proteins (Figure 1B). The pLMG965‐1 shares homology with plasmids of *X. citri* pv. *fuscans* strain CFBP6166 (NZ_CP021005) and CFBP6975 (NZ_CP021009) with more than 90% identity.

### *Genome‐based phylogeny and taxonomy of* Xcv *isolates from India and Brazil*

**FIGURE 2:**
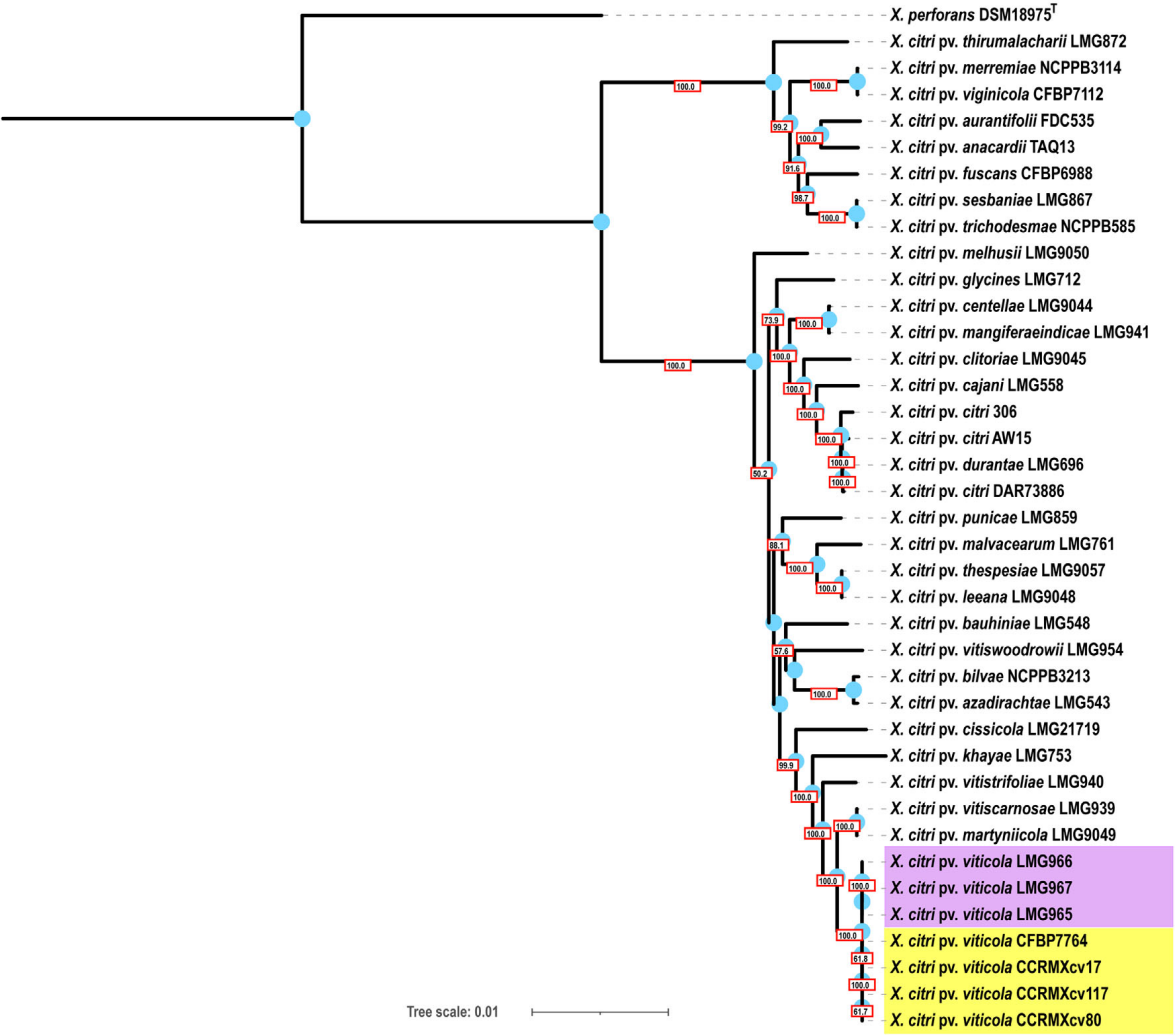
A maximum‐likelihood core gene phylogeny of *X. citri* pathovar representatives. *X. perforans* DSM18975^T^ is taken as an outgroup. Indian *Xcv* strains are depicted in purple‐coloured boxes, and Brazilian *Xcv* strains are depicted in yellow‐coloured boxes. Blue‐coloured circles represent internal nodes, and bootstrap values are mentioned in rectangular boxes at the nodes.

Four genomes of *X. citri* pv. *viticola* reported previously were retrieved from the NCBI GenBank database for comparative genome analysis (de Farias et al., 2020; Ferreira et al., 2019; Lima et al., 2017). We also generated genome sequences of two additional isolates from India. The genome size of LMG966 and LMG967 is 5.11 and 5.10 Mb, with 58 and 59 contigs, respectively. Their GC content is 64%. Detailed genome assembly statistics and metadata of the strains used in the study are given in Table 1. The core gene phylogeny of *Xcv* strains with *X. citri* pathovar representatives revealed that all the *Xcv* strains were forming a separate clade in close association with *X. citri* pv. *martyniicola* LMG9049, *X. citri* pv. *vitiscarnosae* LMG939 and *X. citri* pv. *vitistrifoliae* LMG940 (Figure 2). *X. citri* pv. *vitiscarnosae* LMG939 and *X. citri* pv. *vitistrifoliae* LMG940 infect plants belonging to the Vitaceae family (Grape family) similar to *Xcv* and were also isolated from India during 1961–1962 (Bansal et al., 2017). The monophyletic clade of *Xcv* strains was further forming two subclades, where Indian strains were clustered together in one subclade, and Brazilian strains were clustered in another subclade, indicating further diversification. The *Xcv* strains shared approximately 99.9% orthoANI values with each other and approximately 98.7% orthoANI values with *X. citri* pv. *citri* (*Xcc*) strain 306 (Table S1).

**TABLE 1:** Genome assembly and metadata statistics of the strains used in the study.

Strain	Genome size (Mb)	CDSs	GC content (%)	Coverage	Completeness/contamination	NCBI Accession No.	Geographical location	Isolation year	Reference
LMG965	5.11	4270	64.46	392x	100/0.36	CP084548, CP084549	India	1969	This study
LMG966	5.11	4294	64.45	91x	100/0.36	JALBYO000000000	India	1972	This study
LMG967	5.10	4292	64.45	95x	100/0.36	JALBYN000000000	India	1972	This study
CCRXcv17	5.42	4614	63.90	100x	100/0.36	NZ_PESW01000099	Brazil	2009	(de Farias et al., 2020)
CCRXcv117	5.48	4705	63.70	100x	100/0.36	NZ_PESX01000100	Brazil	2009	(de Farias et al., 2020)
CCRXcv80	5.37	4624	63.80	100x	100/0.36	NZ_NWTJ01000100	Brazil	2009	(Lima et al., 2017)
CFBP7764	5.31	4488	64.30	158x	100/0.36	NZ_PPHE01000001	Brazil	2012	(Ferreira et al., 2019)

### A common frameshift mutation in Xanthomonadin biosynthetic gene cluster

**FIGURE 3:**
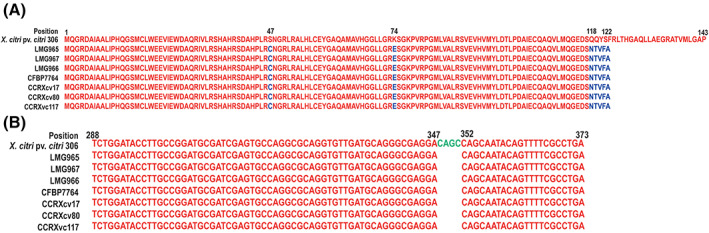
Frameshift mutation location in the *Xanthomonas citri* pv. *viticola* (*Xcv*) strains and *Xcc* 306 (A) Protein sequence alignment of phosphotransferase. Conserved and variable residues are highlighted in red and blue, respectively, and (B) nucleotide sequence alignment. Deleted residues are represented in green colour.

The frameshift mutation in a gene encoding phosphotransferase was reported in the type strain of *Xcv*, LMG965 (Midha & Patil, 2014). Here, we looked for the above‐mentioned frameshift mutation in the rest of the *Xcv* strains from India and Brazil. All other *Xcv* harbour the same frameshift mutation, resulting in a truncated protein of 122 amino acids from 143 amino acids which further revealed the deletion of four nucleotide bases from the 348–351 position (Figure 3). The Xanthomonadin gene cluster analysis revealed that the non‐pigmented colony phenotype had been maintained over the years in the *Xcv* strains from different geographical locations.

### *Genome variation in Indian and Brazilian* Xcv *isolates*

Interestingly, a genome size difference was observed between the Indian and Brazilian strains. Indian strains' genome size is approximately 5.11 Mb, whereas the genome size of Brazilian strains ranges from 5.31 to 5.48 Mb (Table 1). Furthermore, the pangenome analysis revealed the number of core genes for *Xcv* strains was 4164, 86.4% of the total pangenome size (4820 genes). The number of unique genes was very few per strain, LMG965 (19), LMG966 (4), LMG967 (6), CCRXcv80 (67), CCRXcv17 (7), CCRXcv117 (26) and CFBP7764 (71). Furthermore, the unique gene repertoire of Indian strains was compared with that of Brazilian strains. 31 genes were unique to Indian isolates, which encode for type 4 secretion system‐related genes, toxin/antitoxin proteins, hypothetical proteins and recombination/replication‐related proteins. On close inspection, it was found that these genes are associated with the plasmid pLMG965‐1. Hence, genes encoded on the plasmid of LMG965 were present in draft genomes of both the other Indian strains showing that this plasmid is prevalent in Indian isolates and missing or has been lost from the Brazilian population. On the other hand, Brazilian isolates have 47 unique genes, which are mostly encoding CzcABC heavy metal efflux RND transporters, cation transporter, copper resistance‐related genes, transposases and hypothetical proteins. The Brazilian strains carry genes involved in heavy metal resistance and transport that were missing from the Indian isolates. LMG965 and CCRXcv117 were taken as reference genomes to represent the unique gene content of Indian and Brazilian isolates, respectively **(**Table S1 **)**.

### *Acquisition of genomic loci for copper resistance/homeostasis in* Xcv *strains*

**FIGURE 4:**
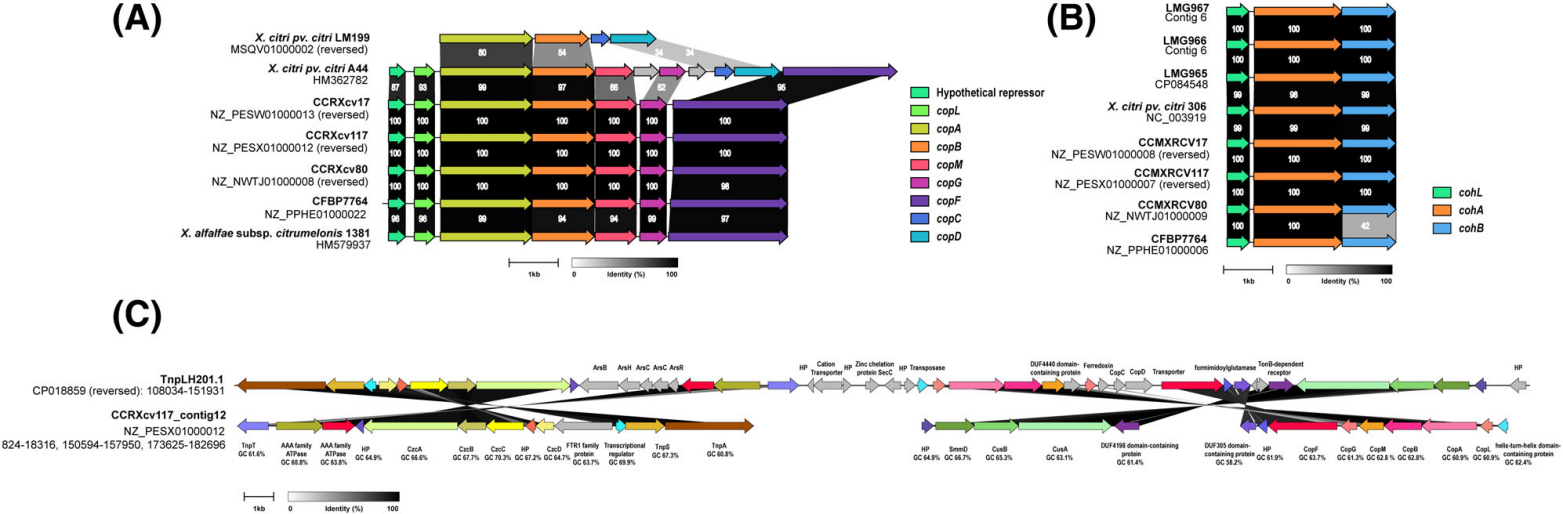
Genomic loci involved in copper resistance/homeostasis gene clusters. (A) Copper resistance operon organisation in the Brazilian *Xanthomonas citri* pv. *viticola* (*Xcv*) strains and related xanthomonads. (B) Copper homeostasis operon organisation in all the *Xcv* strains and *Xcc* 306. (C) Comparison of Tnp201.1 with NZ_PESX01000012 of CCRXv117. Genes are labelled along with their GC content for CCRXcv117. Arrows represent genes. The same genes across the strains are highlighted in the same colour. Numbers mentioned in the connecting regions between genes represent the percentage of amino acid identity. HP denotes hypothetical protein.

The pangenome analysis revealed the presence of copper resistance‐related genes in the Brazilian *Xcv* isolates. The Brazilian *Xcv* strains have an 8128 bp long operon *copLABMGF*, carrying copper resistance‐related genes with a hypothetical repressor upstream of *copL* (Figure 4A). *Xcv* strains show the genetic organisation of copper resistance genes the same as group II (Huang et al., 2021). Many *X. citri* pv. *citri* strains carry group I type copper resistance cluster with the genetic organisation *copLABMGCDF*. *X. citri* pv. *citri* A44, a copper‐resistant strain from Argentina have plasmid‐encoded *copL*, *copA*, *copB*, *copM*, *copG* and *copF* genes, which shared 96%, 97%, 98%, 97%, 64.5% and 97% nucleotide identity with the copper resistance genes of *Xcv* strains while *copC* and *copD* were absent (Table 2) (Behlau et al., 2011; Richard et al., 2017). The hypothetical transcriptional repressor present in *Xcv* strains upstream of *copLABMGCDF* was also found in the A44 strain, along with a transposase and a hypothetical protein (Figure 4A). The *copLABMGCDF* operon was also reported from many other strains of *X. citri* pv. *citri*, *X. gardneri*, *X. vesicatoria* and *X. euvesicatoria* (Richard et al., 2017). A citrus bacterial spot causal organism, *X. alfalfa* subsp. *citrumelonis* 1381 from Florida have the same copper resistance genomic determinants as that of the Brazilian *Xcv* strains, with *copL*, *copA*, *copB*, *copM*, *copG* and *copF* sharing 94%, 96%, 92%, 95%, 98% and 97% nucleotide identity, respectively, and the same hypothetical repressor upstream of *copL* at 95% identity (Behlau et al., 2011). Some other strains, such as *X. axonopodis* pv. *vesicatoria* 7882, *X. citri* pv. *citri* DAR73889 *X. citri* pv. *citri* T4, *X. perforans* LH3, *X. campestris* pv. *campestris* and a human isolate *S. maltophilia* ICU331 also have group II type copper resistance operon (Behlau et al., 2011; Behlau et al., 2017; Huang et al., 2021; Richard et al., 2017; Voloudakis et al., 2005). *X. citri* pv. *citri* LM199 carries a group III type copper cluster, *copABCD* (Huang et al., 2021; Richard et al., 2017). The *copC* and *copD* genes of the *copABCD* operon of LM199 are absent from the Brazilian *Xcv* genomes, while *copA* and *copB* shared 80.5% and 50.4% identity, respectively (Table 2). The *copABCD* type operon is also reported from *X. arboricola* pv. *juglandis* strains (Lee et al., 1994; Richard et al., 2017). Apart from these, three defined groups, a unique chromosomally encoded copper resistance operon from *X. campestris* pv. *vesicatoria* XvP26, along with a two‐component regulatory system CopRS, is not found to be related to copper resistance clusters from the *Xcv* strains (Basim et al., 2005).

**TABLE 2:** BLASTn percentage Identity values between copper resistance genes *copL*, *copA*, *copB*, *copM*, *copG*, and *copF* of the Brazilian *Xcv* strains with previously reported copper resistance operons/extracted from genomes available on NCBI.

Strain	Gene	*Xcv* CCRXcv117 (NZ_PESX01000012)	*Xcv* CCRXcv17 (NZ_PESW01000013)	*Xcv* CCRXcv80 (NZ_NWTJ01000008)	*Xcv* CFBP7764 (NZ_PPHE01000022)
*Xcc* A44	*copL*	96	96	96	96
	*copA*	97	97	97	97
	*copB*	98	98	98	98
	*copM*	97	97	97	97
	*copG* ^a^	64.5	64.5	64.5	64.5
	*copC*	0	0	0	0
	*copD*	0	0	0	0
	*copF*	97	97	97	97
*Sm* ICU331	*copL*	100	100	100	100
	*copA*	99	99	99	99
	*copB*	100	100	100	100
	*copM*	100	100	100	100
	*copG*	99	99	99	99
	*copF*	100	100	100	100
*Xcc* T4	*copL*	99	99	99	99
	*copA*	99	99	99	99
	*copB*	96	96	96	96
	*copM*	97	97	97	97
	*copG*	98	98	98	98
	*copF*	97	97	97	97
*Xp* LH3	*copL*	99	99	99	99
	*copA*	99	99	99	99
	*copB*	96	96	96	96
	*copM*	98	98	98	98
	*copG*	98	98	98	98
	*copF*	97	97	97	97
*Xcc* DAR73889	*copL*	98	98	98	98
	*copA*	99	99	99	99
	*copB*	97	97	97	97
	*copM*	99	99	99	99
	*copG*	98	98	98	98
	*copF*	98	98	98	98
*Xac*1381	*copL*	94	94	94	94
	*copA*	96	96	96	96
	*copB*	92	92	92	92
	*copM*	95	95	95	95
	*copG*	98	98	98	98
	*copF*	97	97	97	97
*Xav*7882	*copL*	95	95	95	95
	*copA*	95	95	95	95
	*copB*	93	93	93	93
	*copM*	93	93	93	93
	*copG*	96	96	96	96
	*copF*	96	96	96	96
*Xcc* LM199	*copA* ^a^	80.5	80.5	80.5	80.5
	*copB* ^a^	50.4	50.4	50.4	50.4
	*copC*	0	0	0	0
	*copD*	0	0	0	0

A*bbreviations*: *Xcc*, *X. citri* pv. *citri*; *Sm*, *Stenotrophomonas maltophilia*; *Xav*, *X. alfalfa* subsp. *citrumelonis X. perforans*; *Xac*, *X. axonopodis* pv. *vesicatoria; Xp, X. perforans*.

^a^ Amino acid identity values are given.

The *SmmD*/*CusAB* gene cluster, which forms a channel through the periplasm (copper/silver efflux pump) present in *S. maltophilia* and reported from pLH201.1 plasmid of *X. citri* pv. *citri* LH201, was also found in the Brazilian *Xcv* strains (Crossman et al., 2008; Richard et al., 2017; Routh et al., 2011). Another important cluster, *czcABCD* involved with the heavy metal (cobalt/zinc/cadmium) efflux associated with the copper‐resistant xanthomonads was found in all the Brazilian *Xcv* strains (Richard et al., 2017) (Figure 4c). These three gene clusters previously described to be encoded on copper‐resistant plasmids are situated on the same contigs in the case of CCRXCV17 and CCRXcv117, whereas they were distributed amongst different contigs in the case of CFBP7764 and CCRXcv80. The GC content of NZ_PESX01000012 of *Xcv* strain CCRXcv117 carrying the heavy metal resistance genes is 60.7%, which is lower than the GC content of the genome (approximately 64%). All the copper resistance and related genes have variable GC content from as low as 58.2%–70.3%. The contig has TnpT, TnpS and TnpA in the vicinity of the *czcABCD* operon, together they constitute an important part of Tn3‐like transposon reported from *Xanthomonas oryzae* (Niu et al., 2015). Previously (Richard et al., 2017), copper‐resistant gene cluster was reported to be associated with Tn3‐like transposase situated on plasmids in *X. citri* pv. *citri* strains. On comparison with the pLH201.1 from *Xcc* LH201 carrying the Tn3 transposon‐associated copper resistance genes, a particular region of the plasmid, TnpLH201.1 (formerly characterised) shares homology with the NZ_PESX01000012 contig of CCRXcv117. CCRXcv117 shows a relationship with the copper resistance genes associated with the TnpLH201.1 region (Figure 4c). However, arsenic resistance‐related genes, as well as a single Ile tRNA gene associated with the TnpLH201.1 that is required for the transcription of the associated genes on the transposon, were absent from CCRXcv117. The conjugative apparatus composed of 16 *tra* genes reported to be associated with the copper‐resistant plasmid, pLH201.1 is absent in the CCRXcv117 (Richard et al., 2017). ICEfinder analysis revealed that region 77,573–139,450 of NZ_PESX01000012 (CCRXcv117) is predicted to be a putative integrative and conjugative element with type 4 secretion system‐related genes. This region is in the vicinity of the copper resistance operon of the CCRXcv117 situated at the same contig.

Interestingly, there is another set of *copLAB* operons reported from some copper‐sensitive *Xanthomonas* strains (Behlau et al., 2011). Apparently, these genes are not involved in copper resistance but rather are associated with copper homeostasis, hence, termed *cohLAB* (Behlau et al., 2011). Homologous of these genes were found in all the *Xcv* strains, both from India and Brazil (Figure 4B). Homologues of *cohL*, *cohA* and *cohB* shared 98.3%, 99.5% and 98.6% nucleotide identity, respectively, with the *cohLAB* operon of *X. citri* pv. *citri* 306. The gene encoding *cohB* carries a frameshift mutation in the CFBP7764. This *cohLAB* operon was situated on different contigs from the *copLABMGF* operon in the *Xcv* strains (Figure 4B). The *cohLAB* of CCRXcv117 shares very less nucleotide identity with *copLAB* genes of the same strain situated on different contig, (*copL*) 32.82%, (*copA*) 66.45% and 46.54% (*copB*).

## DISCUSSION

*Xanthomonas citri* species comprises 30 pathovars, including *X. citri* pv. *viticola* that infects grape plants. The strains belonging to these pathovars are closely related and form monophyletic clades, indicating rapid diversification in a short time (Bansal et al., 2017). The *Xcv* isolates from India and Brazil were forming a distinct monophyletic clade from the rest of the *X. citri* pathovars suggesting their distinct course of evolution. The phylogenetic divergence in the Indian and Brazilian clades is also supported by the increased genome size of the Brazilian isolates when compared to the Indian isolates and the existence of two subclades corresponding to Indian and Brazilian isolates. This can also be attributed to the fact that the Indian isolates are decades old (1969–1972), whereas Brazilian strains were isolated recently (2009–2012). The increased genome size of Brazilian strains hints at the acquisition of important genomic features that might help in the adaptations and survival in different geographic conditions and new agricultural conditions.

The increased genome size of Brazilian strains revealed the acquisition of genomic determinants for copper resistance. As grapes are highly susceptible to fungal and microbial diseases, there is heavy and indiscriminate usage of copper‐based antimicrobials, which might have led to such a rich copper resistance repertoire in Brazilian *Xcv* strains. The *copLABMGF* operon is so far reported to be associated only with the plasmids in the genus *Xanthomonas* (Behlau et al., 2011; Huang et al., 2021; Richard et al., 2017). However, in the case of *Stentrophomonas* strains, it was chromosomally encoded. *S. maltophilia* is found in water, soil, plants and humans, where it might function as a reservoir and carrier for such accessory genes required for survival. The contig carrying *copLABMGF* was carrying the same gene repertoire as that of Tnp201.1, a Tn3‐like transposase from copper‐resistant plasmids in xanthomonads. However, the absence of arsenic resistance‐related genes suggests selective pressure to reduce the cost of maintenance of additional or unrelated accessory genes. Furthermore, the tRNA gene associated with the plasmids carrying TnpLH201.1 was also absent from the *Xcv* strains, similar to the chromosomally integrated transposases (Richard et al., 2017). The 16 *tra* genes apparatus required for the conjugational transfer of copper‐resistant plasmid, pLH201.1 was also absent in the *Xcv* strains (Richard et al., 2017). All these factors, along with the observation of an ICE element in the vicinity of *copLABMGF* operon and other copper‐resistance‐related genes, hint at the possible integration of copper‐resistant genes in the genome of the Brazilian *Xcv* strains.

Till now, Tnp201.1 has been majorly found to be associated with the plasmids in xanthomonads (Richard et al., 2017). However, it was also found to be integrated with the genome of *Stenotrophomonas* sp. LM091, which was isolated from the citrus plant (Richard et al., 2017). The high similarity of copper resistance clusters of *Xcv* strains with plasmid‐borne copper resistance systems, the presence of Tn3‐like transposase, and variable GC content hints at the acquisition of copper resistance genes from other microbes. They could be either integrated in the chromosome or might belong to a separate plasmid unknown until now. Since the analysis was based on the draft genome assemblies, which fail to capture mobile genetic elements such as plasmids, there is no direct evidence that the *copLABMGF* is not, in fact, part of a plasmid in the case of *Xcv* strains. Further, complete genome‐based studies will help in elucidating the origin and the location of these genes.

While copper resistome reflects environmental selection pressure in the *Xcv* strains, another piece of evidence in this regard is the presence of a common frameshift mutation in the Xanthomonadin pigment biosynthetic gene in all the *Xcv* strains. This suggests that the non‐pigmented feature has been maintained over the decades in the *Xcv* strains from different geographical locations. The finding of a common frameshift mutation in a pigment biosynthetic gene cluster indicates the unique evolution of the pathogen and possible environmental‐based selection pressure, which might be because of the way grapevines are cultivated as trellis in orchards. Similarly, in *X. citri* pv. *mangiferaeindicae*, the mango pathogen, the pigment cluster is deleted (Midha & Patil, 2014). However, the pigment locus is not investigated in multiple strains of diverse geographic regions to confirm the mutation or deletion of the cluster. Further molecular and genetic studies into the ecology of non‐pigmented *Xanthomonas* are required.

## CONCLUSION

Apart from the complete genome sequence of the reference strain of *Xcv*, multiple genome sequences from India along with Brazil have allowed us to understand the evolutionary pattern of *Xcv*. Interestingly, the geographic‐specific variation is mediated by a plasmid, as can be seen in Indian isolates, and by genome expansion at the chromosome level, as seen in Brazilian isolates. Further characterisation of unique genes at the molecular and functional levels will help in identifying genes required for pathogenicity and virulence. A common frameshift mutation in all the *Xcv* isolates supports the monophyletic origin of the pathovar confirmed through phylogenomic investigation. Acquisition of multiple loci for copper resistance indicates high selection pressure for copper resistance over the course of time and usage of copper‐based antimicrobial compounds. At the same time, the intrinsic nature of copper resistome, unlike plasmid or extrachromosomal in other *Xanthomonas*, strengthens this observation. The findings, along with genomic resources, will allow the development of markers in the identification and management of this important pathovar of a major fruit plant. Furthermore, the comparative genomic study has provided insights into the origin and nature of the mutation, leading to the variant white colony phenotype of *Xcv* apart from its potential for rapid evolution, as seen in the genomic repertoire of resistance mechanisms of copper resistance and homeostasis. Further genetic and functional studies will help in unravelling the copper resistance mechanism of the Brazilian *Xcv* strains.

## AUTHOR CONTRIBUTIONS

**Rekha Rana:** Formal analysis (lead); methodology (lead); project administration (lead); software (lead); validation (lead); writing – original draft (lead); writing – review and editing (lead). **Gagandeep Jaiswal:** Formal analysis (supporting); methodology (supporting); software (supporting); writing – review and editing (supporting). **Kanika Bansal:** Methodology (supporting); writing – review and editing (supporting). **Prabhu B Patil:** Conceptualization (lead); funding acquisition (lead); supervision (lead); writing – original draft (supporting); writing – review and editing (supporting).

## CONFLICT OF INTEREST STATEMENT

None declared.

## Supporting information


**Table S1.** The genes unique to Brazilian and Indian strains with their designated locus tags, start and stop sites and GenBank annotation are given. Average nucleotide identity values amongst the *Xcv* strains and *Xcc* 306 are mentioned. The IS elements and TALE encoding genes predicted in the LMG965 are given in the table.

## Data Availability

The draft genome sequences of LMG966 and LMG967 are deposited in the NCBI GenBank database under the accession numbers JALBYO000000000 and JALBYN000000000, respectively. The complete genome sequence of LMG965 is submitted in the NCBI GenBank database under the accession number CP084548‐CP084549. The genome sequences data can be accessed at https://figshare.com/s/9296b1dddd6c8c38e81b

## References

[emi413164-bib-0001] Bansal, K. , Midha, S. , Kumar, S. & Patil, P.B. (2017) Ecological and evolutionary insights into *Xanthomonas citri* pathovar diversity. Applied and Environmental Microbiology, 83(9), e02993–e02916. 28258140 10.1128/AEM.02993-16 PMC5394309

[emi413164-bib-0002] Basim, H. , Minsavage, G.V. , Stall, R.E. , Wang, J.‐F. , Shanker, S. & Jones, J.B. (2005) Characterization of a unique chromosomal copper resistance gene cluster from *Xanthomonas campestris* pv. *vesicatoria* . Applied and Environmental Microbiology, 71(12), 8284–8291. 16332814 10.1128/AEM.71.12.8284-8291.2005 PMC1317478

[emi413164-bib-0003] Behlau, F. , Canteros, B.I. , Minsavage, G.V. , Jones, J.B. & Graham, J.H. (2011) Molecular characterization of copper resistance genes from *Xanthomonas citri* subsp. *citri* and *Xanthomonas alfalfae* subsp. *citrumelonis* . Applied and Environmental Microbiology, 77(12), 4089–4096. 21515725 10.1128/AEM.03043-10 PMC3131652

[emi413164-bib-0004] Behlau, F. , Gochez, A.M. , Lugo, A.J. , Elibox, W. , Minsavage, G.V. , Potnis, N. et al. (2017) Characterization of a unique copper resistance gene cluster in *Xanthomonas campestris* pv. *campestris* isolated in Trinidad, West Indies. European Journal of Plant Pathology, 147, 671–681.

[emi413164-bib-0005] Chand, R. & Kishun, R. (1990) Outbreak of grapevine bacterial canker disease in India. Vitis, 29(3), 183–188.

[emi413164-bib-0006] Cooksey, D.A. (1990) Genetics of bactericide resistance in plant pathogenic bacteria. Annual Review of Phytopathology, 28, 201–219.

[emi413164-bib-0007] Crossman, L.C. , Gould, V.C. , Dow, J.M. , Vernikos, G.S. , Okazaki, A. , Sebaihia, M. et al. (2008) The complete genome, comparative and functional analysis of *Stenotrophomonas maltophilia* reveals an organism heavily shielded by drug resistance determinants. Genome Biology, 9(4), 1–13. 10.1186/gb-2008-9-4-r74 PMC2643945 18419807

[emi413164-bib-0008] da Gama, M.A.S. , Mariano, R.d.L.R. , da Silva Júnior, W.J. , de Farias, A.R.G. , Barbosa, M.A.G. , Ferreira, M.Á.d.S.V. et al. (2018) Taxonomic repositioning of *Xanthomonas campestris* pv. *viticola* (Nayudu 1972) dye 1978 as *Xanthomonas citri* pv. *viticola* (Nayudu 1972) dye 1978 comb. nov. and emendation of the description of *Xanthomonas citri* pv. *anacardii* to include pigmented isolates pathogenic to cashew plant. Phytopathology, 108(10), 1143–1153. 29688131 10.1094/PHYTO-02-18-0037-R

[emi413164-bib-0009] da Silva, A.R. , Ferro, J.A. , Reinach, F. , Farah, C. , Furlan, L. , Quaggio, R. et al. (2002) Comparison of the genomes of two Xanthomonas pathogens with differing host specificities. Nature, 417(6887), 459–463. 12024217 10.1038/417459a

[emi413164-bib-0010] de Farias, A.R.G. , da Silva, W.J. , do Nascimento, J.B. , Balbino, V.d.Q. , Benko‐Iseppon, A.M. , Lima, N.B. et al. (2020) Comparative genome analysis and phylogenomic of *Xanthomonas citri* pv. *viticola* lead to new taxonomic insights about species of Xanthomonas.

[emi413164-bib-0011] Ferreira, M.A. , Bonneau, S. , Briand, M. , Cesbron, S. , Portier, P. , Darrasse, A. et al. (2019) *Xanthomonas citri* pv. *viticola* affecting grapevine in Brazil: Emergence of a successful monomorphic pathogen. Frontiers in Plant Science, 10, 489. 31057588 10.3389/fpls.2019.00489 PMC6482255

[emi413164-bib-0012] Gilchrist, C.L. & Chooi, Y.‐H. (2021) Clinker & clustermap. js: automatic generation of gene cluster comparison figures. Bioinformatics, 37(16), 2473–2475. 33459763 10.1093/bioinformatics/btab007

[emi413164-bib-0013] Giovanardi, D. , Bonneau, S. , Gironde, S. , Saux, M.F.‐L. , Manceau, C. & Stefani, E. (2016) Morphological and genotypic features of *Xanthomonas arboricola* pv. juglandis populations from walnut groves in Romagna region, Italy. European Journal of Plant Pathology, 145(1), 1–16.

[emi413164-bib-0014] Grant, J.R. , Arantes, A.S. & Stothard, P. (2012) Comparing thousands of circular genomes using the CGView Comparison Tool. BMC Genomics, 13(1), 1–8. 22621371 10.1186/1471-2164-13-202 PMC3469350

[emi413164-bib-0015] Grau, J. , Reschke, M. , Erkes, A. , Streubel, J. , Morgan, R.D. , Wilson, G.G. et al. (2016) AnnoTALE: bioinformatics tools for identification, annotation and nomenclature of TALEs from Xanthomonas genomic sequences. Scientific Reports, 6(1), 1–12. 26876161 10.1038/srep21077 PMC4753510

[emi413164-bib-0016] Guindon, S. , Dufayard, J.‐F. , Lefort, V. , Anisimova, M. , Hordijk, W. & Gascuel, O. (2010) New algorithms and methods to estimate maximum‐likelihood phylogenies: assessing the performance of PhyML 3.0. Systematic Biology, 59(3), 307–321. 20525638 10.1093/sysbio/syq010

[emi413164-bib-0017] Gurevich, A. , Saveliev, V. , Vyahhi, N. & Tesler, G. (2013) QUAST: quality assessment tool for genome assemblies. Bioinformatics, 29(8), 1072–1075. 23422339 10.1093/bioinformatics/btt086 PMC3624806

[emi413164-bib-0018] He, Y.‐W. , Cao, X.‐Q. & Poplawsky, A.R. (2020) Chemical structure, biological roles, biosynthesis and regulation of the yellow xanthomonadin pigments in the phytopathogenic genus Xanthomonas. Molecular Plant‐Microbe Interactions, 33(5), 705–714. 32027580 10.1094/MPMI-11-19-0326-CR

[emi413164-bib-0019] Health, E.P.o.P. , Bragard, C. , Di Serio, F. , Gonthier, P. , Jaques Miret, J.A. , Justesen, A.F. et al. (2021) Pest categorisation of *Xanthomonas citri* pv. *viticola* . EFSA Journal, 19(12), e06929. 34963789 10.2903/j.efsa.2021.6929 PMC8675326

[emi413164-bib-0020] Huang, C.‐J. , Wu, T.‐L. , Zheng, P.‐X. , Ou, J.‐Y. , Ni, H.‐F. & Lin, Y.‐C. (2021) Comparative genomic analysis uncovered evolution of pathogenicity factors, horizontal gene transfer events, and heavy metal resistance traits in citrus canker bacterium *Xanthomonas citri* subsp. citri. Frontiers in Microbiology, 12, 731711. 34557177 10.3389/fmicb.2021.731711 PMC8453159

[emi413164-bib-0021] Kumar, S. , Stecher, G. , Li, M. , Knyaz, C. & Tamura, K. (2018) MEGA X: molecular evolutionary genetics analysis across computing platforms. Molecular Biology and Evolution, 35(6), 1547–1549. 29722887 10.1093/molbev/msy096 PMC5967553

[emi413164-bib-0022] Lee, I. , Kim, Y.O. , Park, S.‐C. & Chun, J. (2016) OrthoANI: an improved algorithm and software for calculating average nucleotide identity. International Journal of Systematic and Evolutionary Microbiology, 66(2), 1100–1103. 26585518 10.1099/ijsem.0.000760

[emi413164-bib-0023] Lee, Y. , Hendson, M. , Panopoulos, N. & Schroth, M. (1994) Molecular cloning, chromosomal mapping, and sequence analysis of copper resistance genes from Xanthomonas campestris pv. juglandis: homology with small blue copper proteins and multicopper oxidase. Journal of Bacteriology, 176(1), 173–188. 8282694 10.1128/jb.176.1.173-188.1994 PMC205029

[emi413164-bib-0024] Letunic, I. & Bork, P. (2021) Interactive Tree Of Life (iTOL) v5: an online tool for phylogenetic tree display and annotation. Nucleic Acids Research, 49(W1), W293–W296. 33885785 10.1093/nar/gkab301 PMC8265157

[emi413164-bib-0025] Lima, N.B. , Gama, M.A. , Mariano, R.L. , Silva, W.J., Jr. , Farias, A.R. , Falcão, R.M. et al. (2017) Complete genome sequence of Xanthomonas campestris pv. *viticola* strain CCRMXCV 80 from Brazil. Genome Announcements, 5(46), e01263–e01217. 29146856 10.1128/genomeA.01263-17 PMC5690333

[emi413164-bib-0026] Malavolta, V., Jr. , Almeida, I. , Sugimori, M. , Ribeiro, I. , Rodrigues Neto, J. , Pires, E. et al. (1999) Occurrence of Xanthomonas campestris pv. *viticola* in grape in Brazil. Summa Phytopathologica, 25(3), 262–264.

[emi413164-bib-0027] Marques, E. , Uesugi, C.H. & Ferreira, M.A. (2009) Sensitivity to copper in Xanthomonas campestris pv. *viticola* . Tropical Plant Pathology, 34, 406–411.

[emi413164-bib-0028] Midha, S. & Patil, P.B. (2014) Genomic insights into the evolutionary origin of *Xanthomonas axonopodis* pv. citri and its ecological relatives. Applied and Environmental Microbiology, 80(20), 6266–6279. 25085494 10.1128/AEM.01654-14 PMC4178650

[emi413164-bib-0029] Nayudu, M. (1972) Pseudomonas *viticola* sp. nov., incitant of a new bacterial disease of grape vine. Journal of Phytopathology, 73(2), 183–186.

[emi413164-bib-0030] Niu, X.‐N. , Wei, Z.‐Q. , Zou, H.‐F. , Xie, G.‐G. , Wu, F. , Li, K.‐J. et al. (2015) Complete sequence and detailed analysis of the first indigenous plasmid from Xanthomonas oryzae pv. oryzicola. BMC Microbiology, 15(1), 1–15. 26498126 10.1186/s12866-015-0562-x PMC4619425

[emi413164-bib-0031] Page, A.J. , Cummins, C.A. , Hunt, M. , Wong, V.K. , Reuter, S. , Holden, M.T. et al. (2015) Roary: rapid large‐scale prokaryote pan genome analysis. Bioinformatics, 31(22), 3691–3693. 26198102 10.1093/bioinformatics/btv421 PMC4817141

[emi413164-bib-0032] Parks, D.H. , Imelfort, M. , Skennerton, C.T. , Hugenholtz, P. & Tyson, G.W. (2015) CheckM: assessing the quality of microbial genomes recovered from isolates, single cells, and metagenomes. Genome Research, 25(7), 1043–1055. 25977477 10.1101/gr.186072.114 PMC4484387

[emi413164-bib-0033] Peixoto, A.R. , Mariano, R.L. , Moreira, J.O.T. & Viana, I.O. (2007) Alternative hosts of Xanthomonas campestris pv v*iticola* . Fitopatologia Brasileira, 32(2), 161–164.

[emi413164-bib-0034] Prjibelski, A. , Antipov, D. , Meleshko, D. , Lapidus, A. & Korobeynikov, A. (2020) Using SPAdes de novo assembler. Current Protocols in Bioinformatics, 70(1), e102. 32559359 10.1002/cpbi.102

[emi413164-bib-0035] Rana, R. , Bansal, K. , Kaur, A. & Patil, P.B. (2022) Genome dynamics mediated by repetitive and mobile elements in *Xanthomonas citri* pv. durantae. Access Microbiology, 4(10), 000415. 10.1099/acmi.0.000415 PMC9675179 36415734

[emi413164-bib-0036] Richard, D. , Ravigné, V. , Rieux, A. , Facon, B. , Boyer, C. , Boyer, K. et al. (2017) Adaptation of genetically monomorphic bacteria: evolution of copper resistance through multiple horizontal gene transfers of complex and versatile mobile genetic elements. Molecular Ecology, 26(7), 2131–2149. 28101896 10.1111/mec.14007

[emi413164-bib-0037] Routh, M.D. , Zalucki, Y. , Su, C.‐C. , Long, F. , Zhang, Q. , Shafer, W.M. et al. (2011) Efflux pumps of the resistance‐nodulation‐division family: a perspective of their structure, function and regulation in gram‐negative bacteria. Advances in Enzymology and Related Areas of Molecular Biology, 77, 109–146. 21692368 10.1002/9780470920541.ch3 PMC4390553

[emi413164-bib-0038] Seemann, T. (2014) Prokka: rapid prokaryotic genome annotation. Bioinformatics, 30(14), 2068–2069. 24642063 10.1093/bioinformatics/btu153

[emi413164-bib-0039] Trindade, L.C.d. , Marques, E. , Lopes, D.B. & Ferreira, M.Á.d.S.V. (2007) Development of a molecular method for detection and identification of *Xanthomonas campestris* pv. *viticola* . Summa Phytopathologica, 33(1), 16–23.

[emi413164-bib-0040] Varani, A.M. , Siguier, P. , Gourbeyre, E. , Charneau, V. & Chandler, M. (2011) ISsaga is an ensemble of web‐based methods for high throughput identification and semi‐automatic annotation of insertion sequences in prokaryotic genomes. Genome Biology, 12(3), 1–9. 10.1186/gb-2011-12-3-r30 PMC3129680 21443786

[emi413164-bib-0041] Voloudakis, A.E. , Reignier, T.M. & Cooksey, D.A. (2005) Regulation of resistance to copper in Xanthomonas axonopodis pv. vesicatoria. Applied and Environmental Microbiology, 71(2), 782–789. 15691931 10.1128/AEM.71.2.782-789.2005 PMC546827

[emi413164-bib-0042] Walker, B.J. , Abeel, T. , Shea, T. , Priest, M. , Abouelliel, A. , Sakthikumar, S. et al. (2014) Pilon: an integrated tool for comprehensive microbial variant detection and genome assembly improvement. PLoS One, 9(11), e112963. 25409509 10.1371/journal.pone.0112963 PMC4237348

[emi413164-bib-0043] Wick, R.R. , Judd, L.M. , Gorrie, C.L. & Holt, K.E. (2017) Unicycler: resolving bacterial genome assemblies from short and long sequencing reads. PLoS Computational Biology, 13(6), e1005595. 28594827 10.1371/journal.pcbi.1005595 PMC5481147

[emi413164-bib-0044] Xiaojing, F. , Saleem, T. & Huasong, Z. (2022) Copper resistance mechanisms in plant pathogenic bacteria. Phytopathologia Mediterranea, 61(1), 129–138.

